# Supplementary material for: Cytoskeletal components can turn wall-less spherical bacteria into kinking helices
Source: Nat Commun. 2022 Nov 14;13:6930. doi: 10.1038/s41467-022-34478-0 (PMC9663586; doi:10.1038/s41467-022-34478-0)
Supplement: Supplementary file 1 — Supplementary Information [file 41467_2022_34478_MOESM1_ESM.pdf]

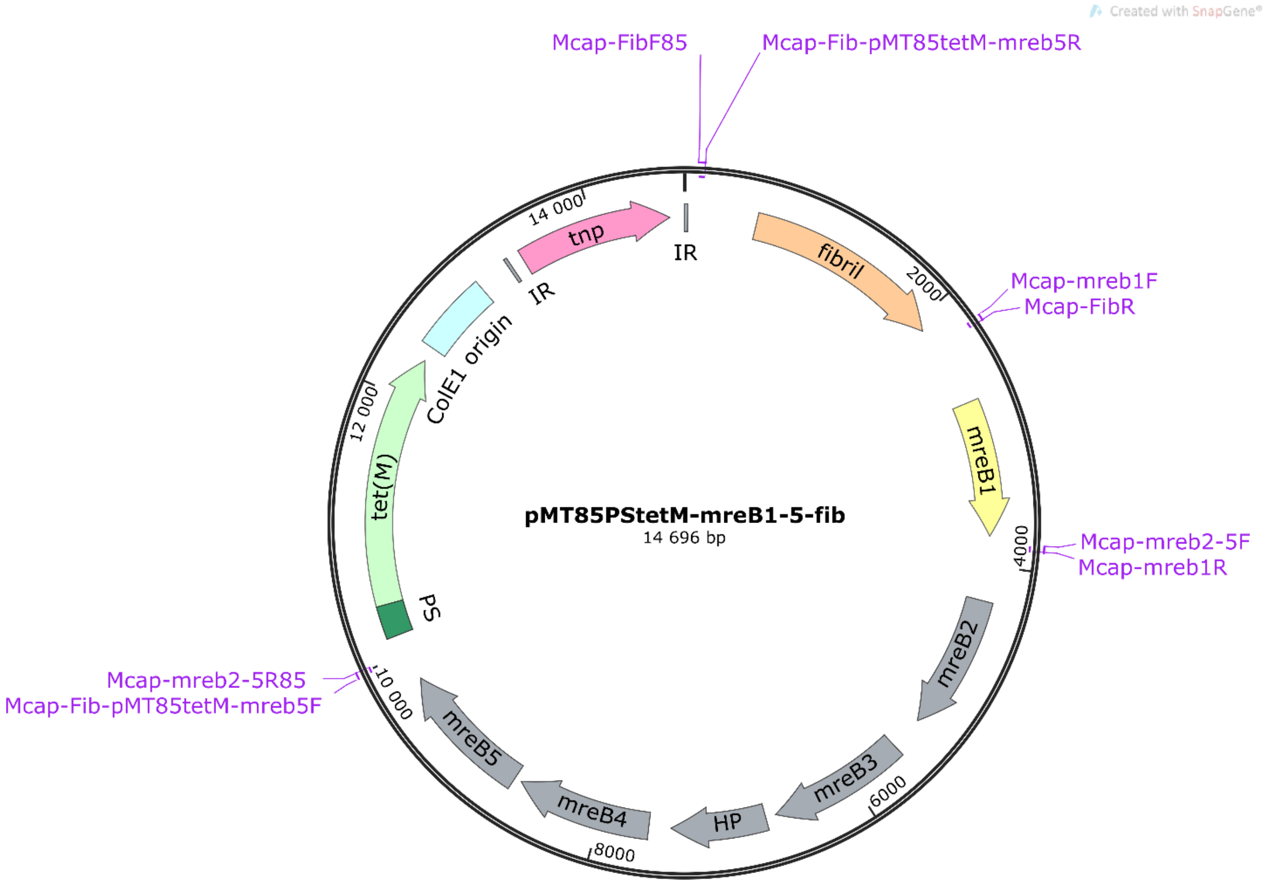

Supplementary Figure 1. Map of the pMT85PStetM-mreB1-5-fib plasmid. This plasmid derives from the transposon based-plasmid pMT85-PStetM (4.82 kbp) which harbors the *tet*(M) gene (from *tn916*) under the control of the spiralin promoter (PS), a ColE1 origin and a transposase encoding gene (*tnp*) flancked by two inverted repeats (IR) from *tn4001* transposon. This plasmid contains 7 others genes (*fibril*, *mreB1*, *mreB2*, *mreB3*, an hypothetical protein encoding gene (HP), *mreB4* and *mreB5*) under the control of their native promoters. Primers used to assemble the plasmid are indicated in pink.

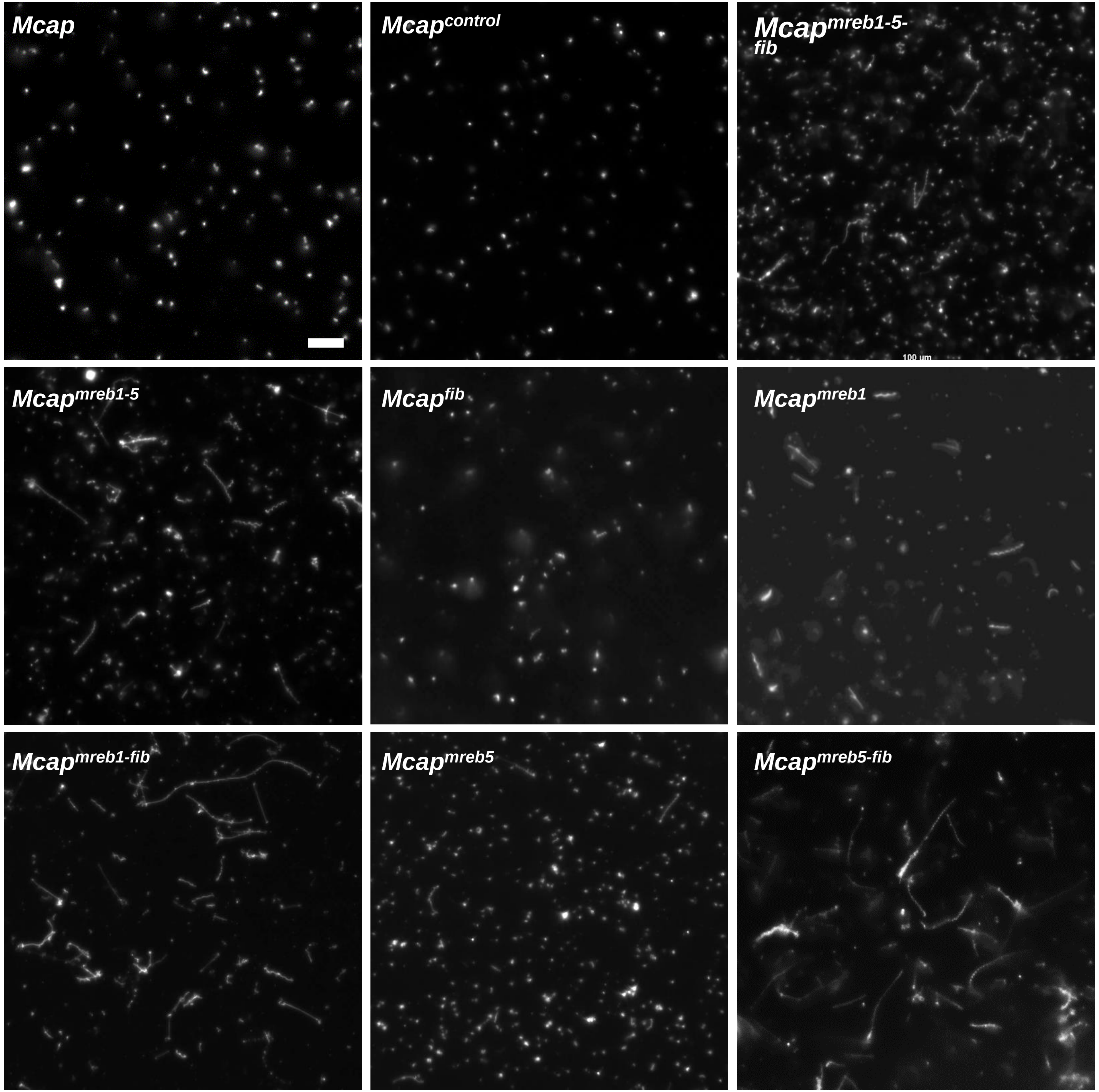

Supplementary Figure 2. Representative darkfield microscopy field images of *Mcap* transformed with different *mreB* and *fib* gene combinations. *Mcap* corresponds to *Mcap* before being transformed, and *Mcap*^{control} corresponds to *Mcap* transformed with the pMT85-PStetM vector without any additional gene. The results are representative of at least three independent analyses. Scale bar: 10 µm.

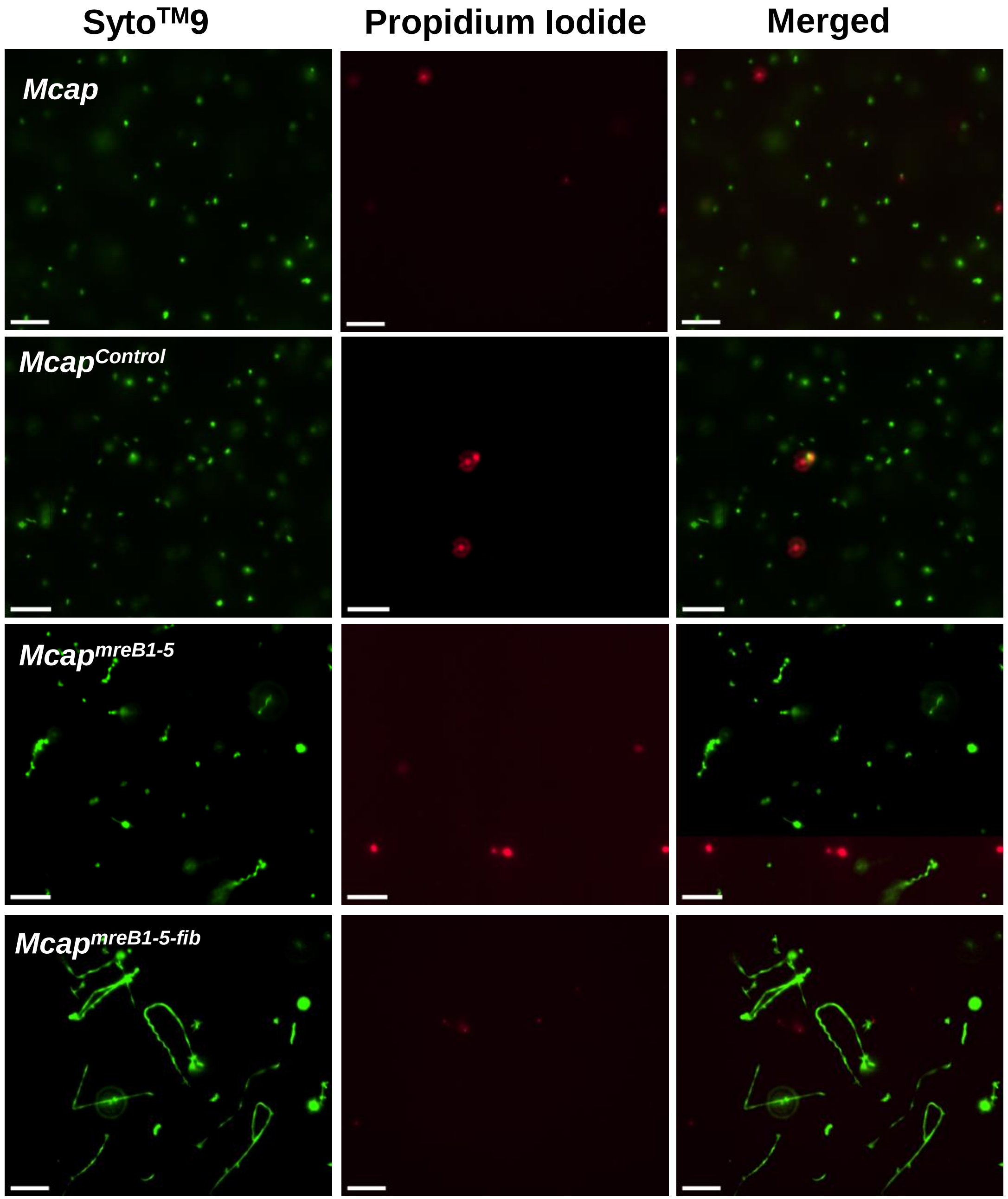

Supplementary Figure 3. Live/Dead staining: Representative fluorescence microscopy images of *Mcap* transformed with different *mreB* and *fib* gene combinations. *Mcap* corresponds to *Mcap* before being transformed, and *Mcap*^{control} corresponds to *Mcap* transformed with the pMT85-PStetM vector without any additional gene. Bacteria were stained using SYTO^{TM}9 to stain live cells green and propidium iodide (PI, middle) to stain dead cells red. Merged images (right) showing bacteria stained with both SYTO-9 and PI are also shown, and a yellow or red merged color was observed for dead cells. Transformation of *Mcap* with *mreB* and *fib* genes induced filamentation and branching in many cells, which were still viable and multiplying at time of observation. The results are representative of at least three independent analyses.

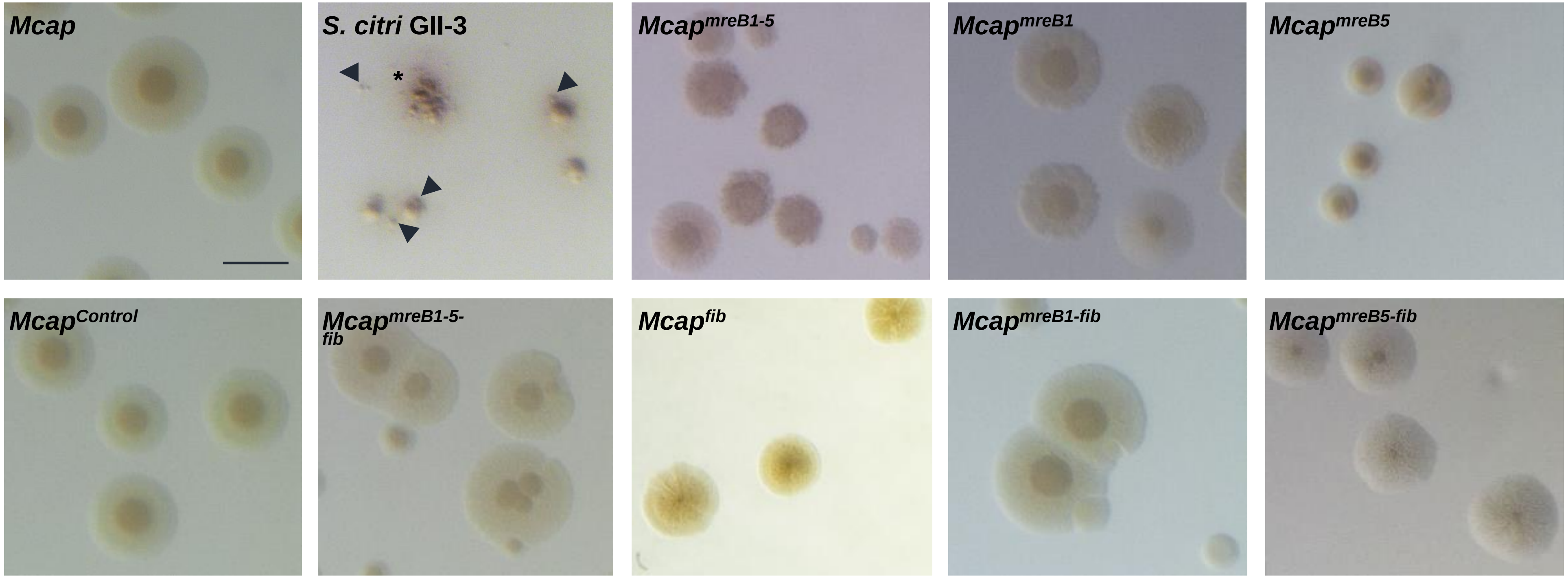

Supplementary Figure 4. **Light microscopy images of colonies of *S. citri* GII-3 cells and of *Mcap* transformed with different *mreB* and *fib* gene combinations**. *Mcap* corresponds to *Mcap* before being transformed, and *Mcap*^{control} corresponds to *Mcap* transformed with the pMT85-PStetM vector without any additional gene. Typical diffuse colonies were observed for *S. citri* with satellite colonies (asterisk points toward a diffuse colony for which the mother colony is not clearly visible anymore; the other colonies are made of a mother colony surrounded by small-sized satellite colonies pointed out by black arrows), and *Mcap*^{control} produced fried-egg colonies. *Mcap* transformants did not form satellite colonies, and those having *mreB5* gene produced darker and smaller colonies. Irregular contour and/or granular aspect were observed for most *Mcap* transformants. The results are representative of at least three independent analyses. Scale bar: 200 µm.

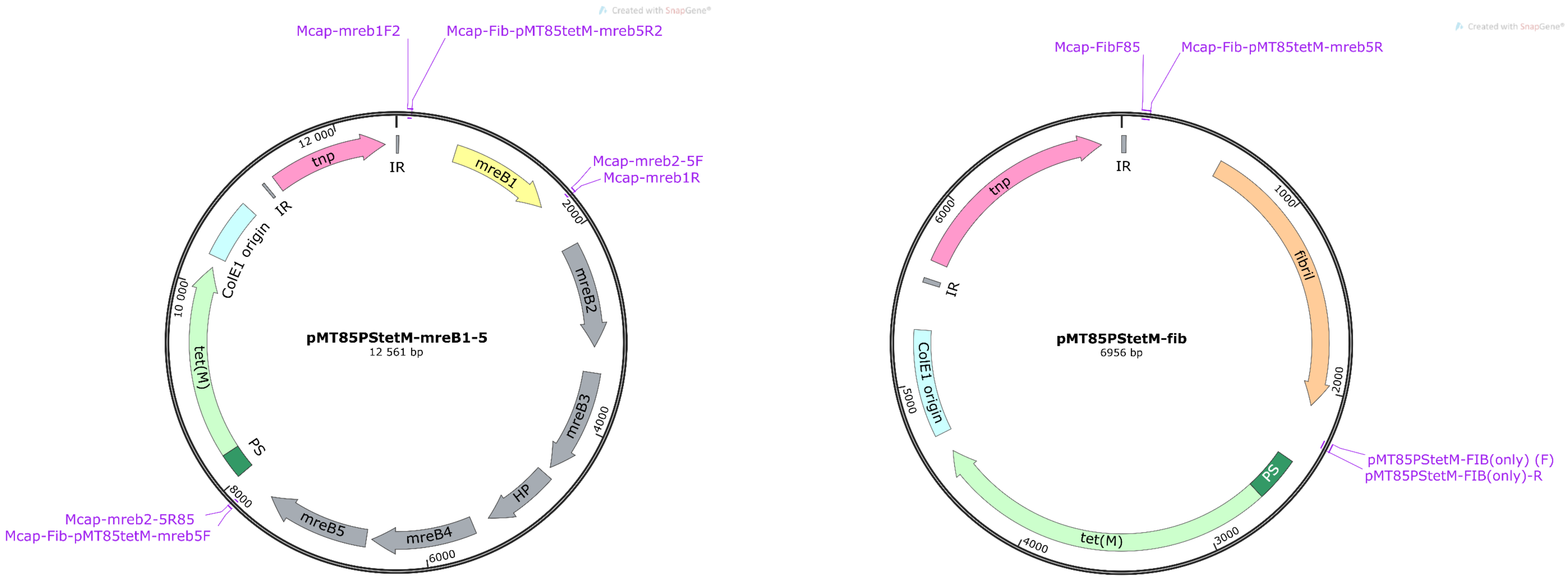

Supplementary Figure 5. Maps of the pMT85PStetM-mreB1-5 and pMT85PStetM-fib plasmids. Both plasmids derive from the transposon based-plasmid pMT85-PStetM (4.82 kbp) which harbors the *tet*(M) gene (from *tn916*) under the control of the spiralin promoter (PS), a ColE1 origin and a transposase encoding gene (*tnp*) flancked by two inverted repeats (IR) from *tn4001* transposon. The pMT85PStetM-mreB1-5 plasmid contains 6 others genes (*mreB1*, *mreB2*, *mreB3*, an hypothetical protein encoding gene (HP), *mreB4* and *mreB5*) under the control of their native promoters while pMT85PStetM-fib plasmid harbors solely the *fibril* encoding gene under the control of its native promoter. Primers used to assemble both plasmids are indicated in pink.

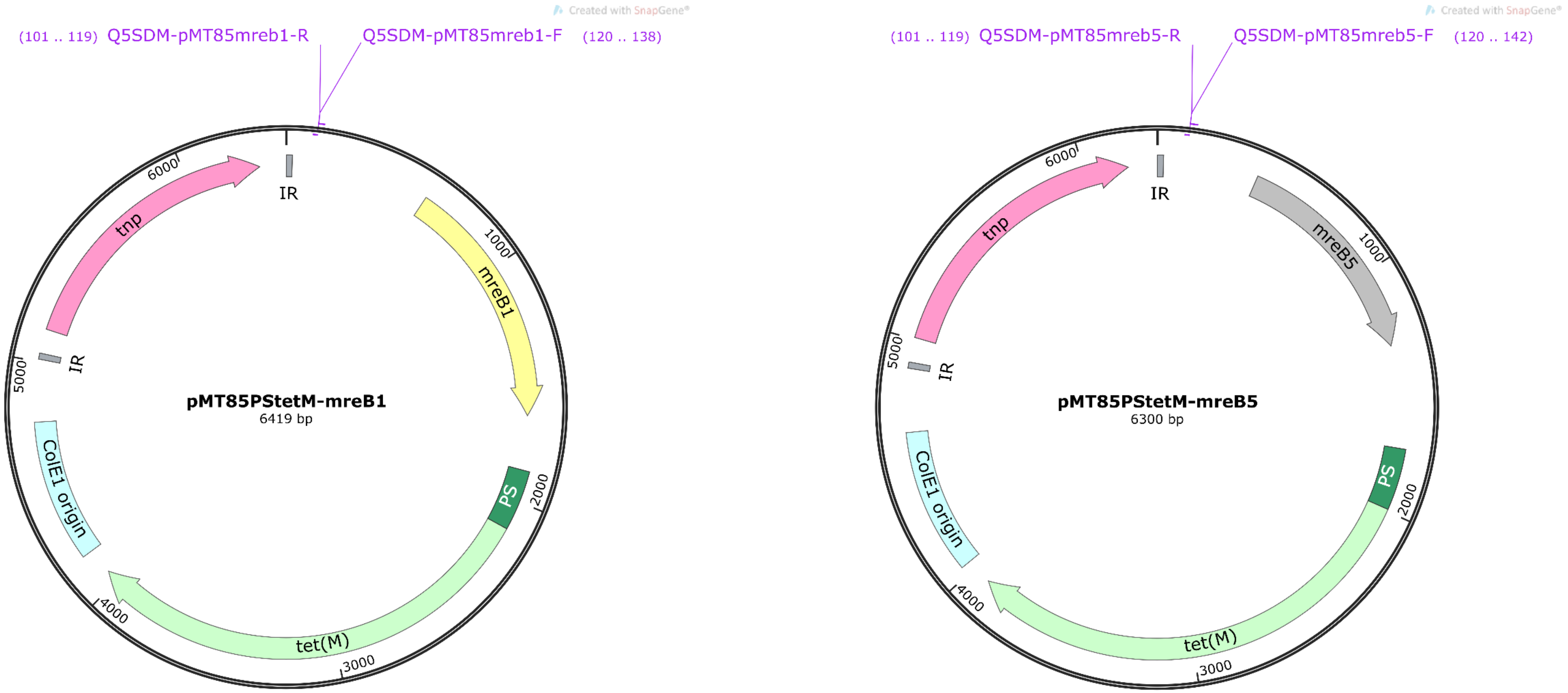

Supplementary Figure 6. Maps of the pMT85PStetM-mreB1 and pMT85PStetM-mreB5 plasmids. Both plasmids derive from the transposon based-plasmid pMT85-PStetM (4.82 kbp) which harbors the *tet*(M) gene (from *tn916*) under the control of the spiralin promoter (PS), a ColE1 origin and a transposase encoding gene (*tnp*) flancked by two inverted repeats (IR) from *tn4001* transposon. The pMT85PStetM-mreB1 and pMT85PStetM-mreB5 contain the *mreB1* and *mreB5* encoding genes respectively, under the control of their native promoters. Primers used to assemble both plasmids are indicated in pink.

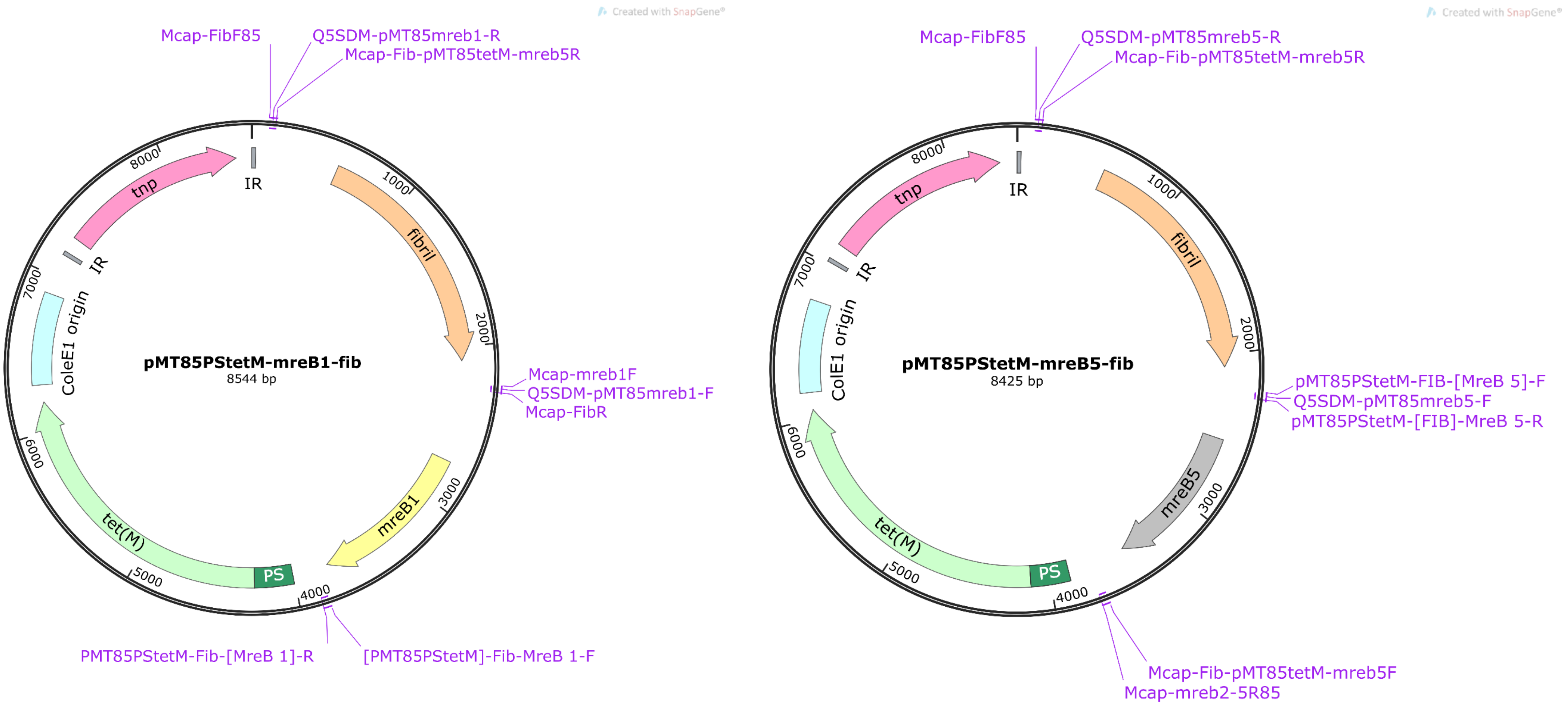

Supplementary Figure 7. Maps of the pMT85PStetM-mreB1-fib and pMT85PStetM-mreB5-fib plasmids. Both plasmids derive from the transposon based-plasmid pMT85-PStetM (4.82 kbp) which harbors the *tet*(M) gene (from *tn916*) under the control of the spiralin promoter (PS), a ColE1 origin and a transposase encoding gene (*tnp*) flancked by two inverted repeats (IR) from *tn4001* transposon. The pMT85PStetM-mreB1-fib harbors the *mreB1* and *fib* encoding genes under the control of their native promoters while the pMT85PStetM-mreB5 harbors the *mreb5* and *fib* encoding genes under the control of their native promoters. Primers used to assemble both plasmids are indicated in pink.

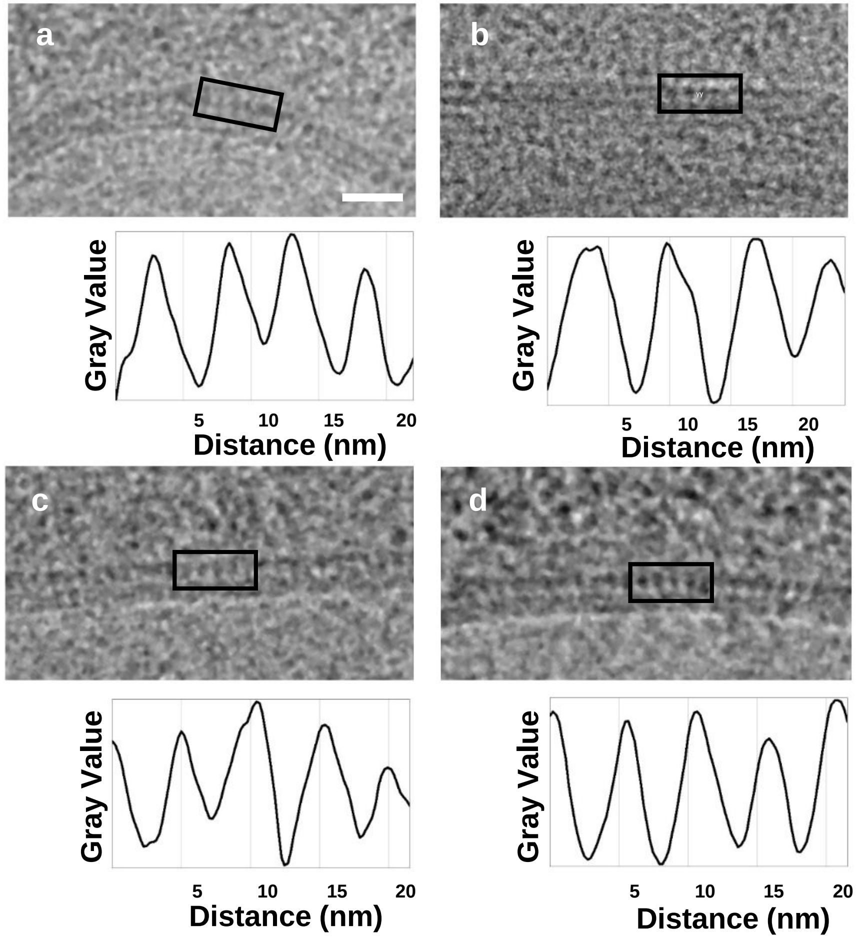

Supplementary Figure 8. **Mean distance of repeats observed in *S. citri* (a), *Mcap*$^{mreB1\text{-}5\text{-}fib}$ (b) *Mcap*$^{mreB1\text{-}5}$ (c) and *Mcap*$^{mreB5\text{-}fib}$ (d).** Measurements were made from cryo-electron microscopy images (see Fig. 4 of the main text) using the function ‘plot profile’ of ImageJ software applied to area outlined in black. Mean distances were 5.2+/-0.7 nm for *S. citri* (a), 6.3+/-0.9 nm for *Mcap*$^{mreB1\text{-}5\text{-}fib}$ (b), 5.2+/-0.4 nm for *Mcap*$^{mreB1\text{-}5}$ (c), and 5.1+/-0.6 nm for *Mcap*$^{mreB5\text{-}fib}$ (d). The results are representative of at least two independent analyses. Scale bar: 20 nm.

Supplementary Table 1. Localisation of the added genes in the *Mcap* genome

Plasmids	Clone number	Integration site	Position in the *Mcap* genome (nt)
pMT85-PStetM	2.1	Mcap0559 - TraG/TraD	690/2001
pMT85-PStetM	5.1	Mcap0466 - Endopeptidase O	236/1893
pMT85PStetM-mreB1-5-fib	8.7	Mcap0222 - Threonyl tRNA synthetase	859/1160
pMT85PStetM-mreB1-5-fib	32.1	Mcap0846 - Lipoprotein	1031/2091
pMT85PStetM-mreB1-5	7.5	Complete integration of the plasmid in the *Mcap* genome	NA
pMT85-PStetM-fib	3.1	Mcap0086 - Lipoprotein	972/2091
pMT85-PStetM-fib	3.2	Intergenic region between Mcap0791 (HP) and Mcap0792 (DNA Topoisomerase)	913795/1010023
pMT85-PStetM-fib	8.4	Mcap0791 - HP	157/876
pMT85-PStetM-fib	3.22	Mcap0460 - 6 Phospo gluconate dehydrogenase	278/900
pMT85-PStetM-mreB5	12.1	Complete integration of the plasmid in the *Mcap* genome	NA
pMT85-PStetM-mreB5	24.3	Complete integration of the plasmid in the *Mcap* genome	NA
pMT85-PStetM-mreB5-fib	17.1	Complete integration of the plasmid in the *Mcap* genome	NA
pMT85-PStetM-mreB5-fib	17.3	Mcap0362 - HP	1900/2508
pMT85-PStetM-mreB1	8.1	Complete integration of the plasmid in the *Mcap* genome	NA
pMT85-PStetM-mreB1	7.1	Complete integration of the plasmid in the *Mcap* genome	NA
pMT85-PStetM-mreB1-fib	13.1	Complete integration of the plasmid in the *Mcap* genome	NA

NA: not applicable. Primers used during SPP allowed concluding that the entire transposon-based plasmid integrated the *Mcap* genome in these clones. However, they do not allow determining the insertion site of the transposon-based plasmid.

Supplementary Table 2. Normalized abundance in percentage of MreBs and Fib in *S. citri* at different times of growth

	MreB1	MreB2	MreB3	MreB4	MreB5	Fib
***S. citri* (pH=7.35)[(1)]**	**0.36**	**0.34**	**0.41**	**0.84**	**1.09**	**0.73**
***S. citri* (pH=7.25)**	**0.30**	**0.26**	**0.51**	**0.68**	**0.88**	**0.79**
***S. citri* (pH=7.02)**	**0.29**	**0.28**	**0.45**	**0.54**	**1.07**	**0.60**
***S. citri* (pH=6.71)**	**0.35**	**0.27**	**0.47**	**0.64**	**1.05**	**0.75**
***S. citri* (pH=6.5)**	**0.39**	**0.26**	**0.46**	**0.71**	**1.26**	**0.75**
**Mean of all times of growth**	**0.34±0.04**	**0.28±0.03**	**0.46±0.04**	**0.68±0.11**	**1.07±0.13**	**0.73±0.07**

[(1)]The mean of normalized abundances (in %) was calculated to show the absence of abundance variation during *S. citri* growth. Spiroplasmas were harvested at different times of growth in SP4 medium, corresponding to different pH of the medium.

Supplementary Table 3. ***Mcap*** **transformation efficiency with the plasmids built during this study and harboring different combination of** ***mreB*** **and** ***fibril*** **encoding gene(s)**

**Plasmid**	**Transformation efficiencies***<br>**(Number tfs/mL/µg of plasmids)**
pMT85-PStetM	3,15E-08
pMT85PStetM-mreB1-5-fib	1,85E-10
pMT85PStetM-mreB1-5	7,21E-11
pMT85-PStetM-fib	1,85E-08
pMT85-PStetM-mreB5	2,44E-11
pMT85-PStetM-mreB5-fib	2,00E-10
pMT85-PStetM-mreB1	1,20E-10
pMT85-PStetM-mreB1-fib	1,70E-10

*Calculated from at least 3 independent experiments

Supplementary Table 4. DNA cassettes used for plasmids constructions

**Plasmids Names**	**Number of DNA cassettes used for the Gibson assembly reaction**	**Cassette "pMT85PStetM"** (~4,862bp)	**Cassette "*fibril*"** SPICI12_006 (~2,175bp)	**Cassette "*mreB1*"** (SPICI13_009) (~1,637bp)	**Cassette "*mreB2-3-HP-4-5*"** (SPICI01A_045 to 049) (~6,185bp)
pMT85PStetM-mreB1-5-fib	4	+	+	+	+
pMT85PStetM-mreB1-5	3	+	-	+	+
pMT85PStetM-fib	2	+	+	-	-
pMT85PStetM-mreB1-fib	3	+	+	+	-
pMT85PStetM-mreB5-fib	3	+	+	-	+ *(mreB5 only)*
pMT85PStetM-mreB1	Plasmid derived from pMT85PStetM-mreB1-fib and built using the Q5® Site-Directed Mutagenesis Kit Protocol.				
pMT85PStetM-mreB5	Plasmid derived from pMT85PStetM-mreB5-fib and built using the Q5® Site-Directed Mutagenesis Kit Protocol.				
