# Supplementary material for: Cytoskeletal components can turn wall-less spherical bacteria into kinking helices
Source: Nat Commun. 2022 Nov 14;13:6930. doi: 10.1038/s41467-022-34478-0 (PMC9663586; doi:10.1038/s41467-022-34478-0)
Supplement: Supplementary file 6 — Reporting Summary [file 41467_2022_34478_MOESM6_ESM.pdf]

nature portfolio

Corresponding author(s): Laure Béven
Last updated by author(s): 21/10/22

Reporting Summary

Nature Portfolio wishes to improve the reproducibility of the work that we publish. This form provides structure for consistency and transparency in reporting. For further information on Nature Portfolio policies, see our Editorial Policies and the Editorial Policy Checklist.

Statistics

For all statistical analyses, confirm that the following items are present in the figure legend, table legend, main text, or Methods section.

n/a	Confirmed	
☐	☒	The exact sample size (*n*) for each experimental group/condition, given as a discrete number and unit of measurement
☐	☒	A statement on whether measurements were taken from distinct samples or whether the same sample was measured repeatedly
☐	☒	The statistical test(s) used AND whether they are one- or two-sided<br>*Only common tests should be described solely by name; describe more complex techniques in the Methods section.*
☐	☒	A description of all covariates tested
☒	☐	A description of any assumptions or corrections, such as tests of normality and adjustment for multiple comparisons
☐	☒	A full description of the statistical parameters including central tendency (e.g. means) or other basic estimates (e.g. regression coefficient) AND variation (e.g. standard deviation) or associated estimates of uncertainty (e.g. confidence intervals)
☐	☒	For null hypothesis testing, the test statistic (e.g. *F*, *t*, *r*) with confidence intervals, effect sizes, degrees of freedom and *P* value noted<br>*Give P values as exact values whenever suitable.*
☒	☐	For Bayesian analysis, information on the choice of priors and Markov chain Monte Carlo settings
☒	☐	For hierarchical and complex designs, identification of the appropriate level for tests and full reporting of outcomes
☒	☐	Estimates of effect sizes (e.g. Cohen's *d*, Pearson's *r*), indicating how they were calculated

Our web collection on statistics for biologists contains articles on many of the points above.

Software and code

Policy information about availability of computer code

Data collection	Fluorescence images were acquired using Micro-Manager 2.0.0 open source microscopy software (https://micro-manager.org/Version_2.0). Darkfield microscopy images and videos were acquired using the NIS Elements Br 5.30.03 software (Nikon). Plasmid maps were created using Snapgene(R) .
Data analysis	Morphology parameters measurements were made using the NIS-Elements Br software from Nikon. The brightness and contrast were adjusted using Fiji 2.35. Graphs and statistical calculations were made using Excel 2019. Morphological changes during the propagation of membrane deformations were made using Blender 3.2.2. CryoEM images were processed using ImajeJ 1.4.3.68. The proteomics raw files were analysed with Proteome Discoverer 1.4.1.14 (Thermo Scientific). A database search was performed with the Mascot (version 2.8.0) algorithm. Peptide validation was performed using the Percolator algorithm (https://www.ncbi.nlm.nih.gov/pmc/articles/PMC2710313).

For manuscripts utilizing custom algorithms or software that are central to the research but not yet described in published literature, software must be made available to editors and reviewers. We strongly encourage code deposition in a community repository (e.g. GitHub). See the Nature Portfolio guidelines for submitting code & software for further information.

Data

Policy information about availability of data

All manuscripts must include a data availability statement. This statement should provide the following information, where applicable:

- Accession codes, unique identifiers, or web links for publicly available datasets
- A description of any restrictions on data availability
- For clinical datasets or third party data, please ensure that the statement adheres to our policy

The mass spectrometry data supporting the findings of this study have been deposited to the ProteomeXchange Consortium via the PRIDE partner repository with the data set identifier PXD036290 (http://www.ebi.ac.uk/pride].
All Mycoplasma capricolum subsp. capricolum protein sequences can be found at https://www.ncbi.nlm.nih.gov/search/all/?term=GCF_000012765.1_ASM1276v1_protein. All Spiroplasma citri GII3 protein sequences were previously reported in Carle et al., 2010 (PMID: 20363791). All other data supporting the findings of this study are available within the paper and its supplementary information files.

Human research participants

Policy information about studies involving human research participants and Sex and Gender in Research.

Reporting on sex and gender	Our study did not involve any human research participants. Sex and gender do not apply to bacteria.
Population characteristics	n/a
Recruitment	n/a
Ethics oversight	n/a

Note that full information on the approval of the study protocol must also be provided in the manuscript.

Field-specific reporting

Please select the one below that is the best fit for your research. If you are not sure, read the appropriate sections before making your selection.

[x] Life sciences [ ] Behavioural & social sciences [ ] Ecological, evolutionary & environmental sciences

For a reference copy of the document with all sections, see nature.com/documents/nr-reporting-summary-flat.pdf

Life sciences study design

All studies must disclose on these points even when the disclosure is negative.

Sample size	Sample sizes were similar to those generally employed in the field. For morphology parameters measurements such as those regarding the helical pitch or cell length measurements, the number of cells required to be confident in the applied Student's t-test would be n=30. In order to get stronger statistical values, we increased this number of cells to n=50 (up to n=150). For cryoEM analyses, 2 or 3 samples for each constructs were analyzed and allowed the imaging of the cytoskeleton where the genes of interest were introduced. Around ten fields of cells were observed for each sample. Proteomics have been applied on independent samples, for which the number of passaging in liquid broth after transformation is indicated in the manuscript, and correlated to its morphology.
Data exclusions	Criteria defined for measurements are described in the Methods section. No data was excluded when the sample met these criteria.
Replication	All experiments have been replicated at least twice. All the experiments were highly reproducible.
Randomization	Randomization was not necessary for the experiments. In the case of genomics, transcriptomics and phylogenetics, randomization is not possible. In the case of image analyses, all the bacteria present on the images, and respecting the criteria established in advance, were measured. It was therefore not useful to randomly select cells to be measured.
Blinding	Blind analyses were performed by a second investigator for morphology parameters measurements. For all other experiments, blinding was not necessary.

Reporting for specific materials, systems and methods

We require information from authors about some types of materials, experimental systems and methods used in many studies. Here, indicate whether each material, system or method listed is relevant to your study. If you are not sure if a list item applies to your research, read the appropriate section before selecting a response.

Materials & experimental systems

n/a	Involved in the study
☒	☐ Antibodies
☒	☐ Eukaryotic cell lines
☒	☐ Palaeontology and archaeology
☒	☐ Animals and other organisms
☒	☐ Clinical data
☒	☐ Dual use research of concern

Methods

n/a	Involved in the study
☒	☐ ChIP-seq
☒	☐ Flow cytometry
☒	☐ MRI-based neuroimaging
