# Supplementary material for: Cytoskeletal components can turn wall-less spherical bacteria into kinking helices
Source: Nat Commun. 2022 Nov 14;13:6930. doi: 10.1038/s41467-022-34478-0 (PMC9663586; doi:10.1038/s41467-022-34478-0)
Supplement: Supplementary file 7 — Description of Additional Supplementary Files [file 41467_2022_34478_MOESM7_ESM.pdf]

Description of Additional Supplementary Files:

File name: Supplementary Data 1

List of primers used in this study.

File name: Supplementary Movie 1

S. citri cells have a kink-based motility. *S. citri* cells were grown in SP4 medium and observed using darkfield microscopy. Motility of the helical cells is due to the propagation of a kink, which changes helicity of the cell body. Scale bar: 5 µm. Movie 1 is representative of at least three independent experiments.

File name: Supplementary Movie 2

Mcap cells were transformed with a plasmid carrying *mreB1-5* genes and fibril. The resulting transformants population showed heterogeneity in their morphology. The helical *Mcap*$^{mreB1\text{-}5\text{-}fib}$ transformants can show propagation of kinks along the cell body in SP4 medium, reproducing the kinks observed in *S. citri* cells (sequence 1). In sequence 2 of the movie, a non-kinking helical cell is shown. Bending nonhelical filaments were also observed (sequence 3). Scale bar: 5 µm. Movie 2 is representative of at least three independent experiments.

File name: Supplementary Movie 3

Mcap cells were transformed with a plasmid carrying *mreB1-5* genes (sequence 1). The resulting helical *Mcap*$^{mreB1\text{-}5\text{-}fib}$ transformants could show propagation of kinks along the cell body in SP4 medium, reproducing the kinks observed in *S. citri* cells. The white arrow points toward a kinking helical cell. When *Mcap* was transformed with a plasmid carrying only *mreB5* gene (sequence 2), the resulting transformants could show helicity and kink-like membrane deformations. In contrast to *S. citri*, such *Mcap*^{mreB5} cells did not conserve their helicity during the propagation of the kink-like deformations, which can trigger the formation of entangled filaments. In sequence 2 of the movie, a representative helical cell showing kink-like deformation and transient loss of helicity is circled in red. In sequence 3, a helical *Mcap*^{fib} transformant (red circle) shows helicity, kink-like deformations with transient loss of helicity. Scale bar: 5 µm. Movie 3 is representative of at least three independent experiments.
